# Supplementary material for: Genome-Wide Association Study on the Content of Nucleotide-Related Compounds in Korean Native Chicken Breast Meat
Source: Animals (Basel). 2023 Sep 20;13(18):2966. doi: 10.3390/ani13182966 (PMC10525433; doi:10.3390/ani13182966)
Supplement: Supplementary file 1 [file animals-13-02966-s001.zip › Supplementary Tables S1 and S2.pdf]

Table S1. Number of experimental chickens that make up subgroups separated by generation and sex.

Generation	Male	Female	Total
1st generation	-	158	158
2nd generation	98	94	192
3rd generation	31	65	96
4th generation	99	92	191
**Total**	228	409	637

Table S2. The mean and standard deviation of subgroups′ phenotypic values separated by generation and sex (unit: mM).

Traits	Generation				Sex	
	1st generation	2nd generation	3rd generation	4th generation	Male	Female
Inosine	1.303 ± 0.487	0.896 ± 0.402	1.164 ± 0.395	1.440 ± 0.356	1.226 ± 0.430	1.186 ± 0.483
IMP	3.724 ± 0.891	5.438 ± 0.602	5.880 ± 0.715	5.059 ± 0.553	5.369 ± 0.714	4.741 ± 1.102
Hypoxanthine	0.694 ± 0.224	0.288 ± 0.097	0.269 ± 0.076	0.463 ± 0.161	0.416 ± 0.166	0.451 ± 0.254
